# Supplementary material for: Magnaporthe oryzae Effector AvrPik-D Targets Rice Rubisco Small Subunit OsRBCS4 to Suppress Immunity
Source: Plants (Basel). 2024 Apr 27;13(9):1214. doi: 10.3390/plants13091214 (PMC11085154; doi:10.3390/plants13091214)
Supplement: Supplementary file 1 [file plants-13-01214-s001.zip › plants-2945571-supplementary.pdf]

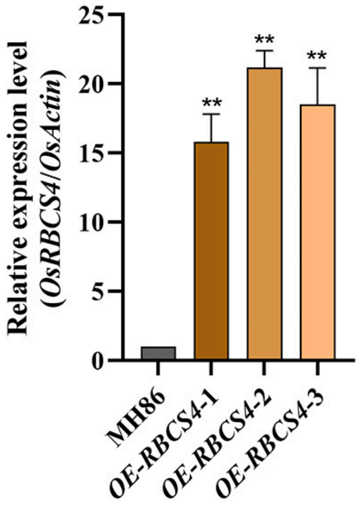

Figure S1 Overexpression of OsRBCS4 in rice validated by qRT-PCR. (A) Expression levels of the OsRBCS4 gene in three overexpression lines were measured by quantitative real-time polymerase chain reaction (qRT-PCR). Each bar represents the mean standard deviation (SD) (n = 3). Asterisks indicate significant differences analyzed by Student's t-test (*P < 0.05, **P < 0.01, ***P < 0.0001).

Supplemental Table 1

List of primers used in the study.

Primer	Sequence (5'--3')	Described
Cluc-OsRBCS4-KpnI-F	tacgcgtcccggggcggtaccATGGCTCCCTCGGTGATGG	For LUI assay
Cluc-OsRBCS4-SalI-R	acgaaagctctgcaggtcgacTTAGTTGCCGCCTGACTCCTC	
pMAL-OsRBCS4-EcoRI-F	gagggaaggatttcagaattcATGGCTCCCTCGGTGATGG	For Pull-down assay
pMAL-OsRBCS4-XbaI-R	tgcctgcaggtcgactctagaTTAGTTGCCGCCTGACTCCTC	
PVX-OsRBCS4-HA-ClaI-F	tcagcaccagctagcatcgatATGGCTCCCTCGGTGATGG	For Co-IP assay
PVX-OsRBCS4-HA-SmaI-R	aatctctagaggatccccgggGTTGCCGCCTGACTCCTCG	
AvrPikD-GFP-Tob-F	aacgatactcgagggggatccATGGAAACGGGCAACAAATATATA	
AvrPikD-GFP-Tob-R	gggaaattcgctagtggatccTCAAAGATCTACCATGTACAGCTCG	
RFP-AvrPik-D-F	aagggatccccgggtgagctcATGGAAACGGGCAACAAATATATA	
RFP-AvrPik-D-R	agcggccgcactagtaagcttTTAAAAGCCGGGCCTTTTTT	For transient expression

223-OsRBCS4-SacIF	gtgggatccccgggtgagctcATGGCTCCCTCGGTGATGG	of rice protoplasts assay
223-OsRBCS4-NotIR	cccttgctcaccatagcggccgcGTTGCCGCCTGACTCCTCG	
OE-RBCS4-F	CGggatccATGGCTCCCTCGGTGATGG	OsRBCS4 overexpression in transgenic rice
OE-RBCS4-R	GGggtaccTTAGTTGCCGCCTGACTCC	
BD-*AvrPik-D*-F	tcagaggaggacctgcatatgATGGAAACGGGCAACAAATATATA	Construct vector for Y2H assay
BD-AvrPik-D-R	gcaggtcgacggatccccgggTTAAAAGCCGGGCCTTTTTT	
AD-RBCS4-F	gtaccagattacgctcatatgATGGCTCCCTCGGTGATGG	
AD-RBCS4-R	ccgtatcgatgcccacccgggTTAGTTGCCGCCTGACTCCTC	
OsUG_F	TTCTGGTCCTTCCACTTTCAG	qRT-PCR of rice genomic ubiquitin
OsUG_R	ACGATTGATTTAACCAGTCCATGA	
MoPot2-F	ACGACCCGTCTTTACTTATTTGG	qRT-PCR of *M.oryzae* retrotransposon Pot2
MoPot2-R	AAGTAGCGTTGGTTTTGTTGGAT	
QRT-OsRbcS4 F1	GCAATGGCGGAAGGATCAGA	qRT-PCR
QRT-OsRbcS4 R1	GGAGCGCGAGAGAACAATTACG	qRT-PCR
OsACTIN1-QRT-F	TGTATGCCAGTGGTCGTACCA	qRT-PCR
OsACTIN1-QRT-R	CCAGCAAGGTCGAGACGAA	qRT-PCR
OsPBZ1-QF	GGTGTGGGAAGCACATAC	qRT-PCR
OsPBZ1-QR	GTCTCCGTCGAGTGTGACTTG	qRT-PCR
OsPR1b-QF	CCCCTCCCAAGCTCAAAACTCC	qRT-PCR
OsPR1b-QR	TGCCCGCACACCTTCCCC	qRT-PCR
OsPAL6-QF	GCTCTTCCGCACACACAACTC	RT-PCR
OsPAL6-QR	TTGCTCGAGCGGTTTGC	RT-PCR
